# Aquablation versus laser enucleation of the prostate for benign prostatic hyperplasia: a systematic review and pairwise meta-analysis of head-to-head studies including the first randomized trial

**DOI:** 10.3389/fsurg.2026.1937749

**Published:** 2026-09-21

**Authors:** Mohammad Zaid Jarai, Amr Elmekresh, Shaikha Alshamsi, Abdalla Aboelkheir, Fadhel Yusuf, Tariq Abdul Hamid, Ammar Agha, Ayman Alyammahi, Thureya Binashour, Murtadha Altammam, Salma Eldesouki, Joanna Jayakumar, Kais Kotiesh, Mohammad Hassan Al Hamad, Rafe Alhayek, Abdulmunem Mohsen, Senthil Subramanian, Salam AlHasani, Fariborz Bagheri, Yaser Saeedi

**Affiliations:** 1 Department of Urology, Dubai Hospital, Dubai Health, Dubai, United Arab Emirates; 2 Department of Urology, Mediclinic Welcare Hospital, Dubai, United Arab Emirates

**Keywords:** Aquablation, benign prostatic hyperplasia, ejaculatory dysfunction, holmium laser enucleation, lower urinary tract symptoms, meta-analysis, minimally invasive surgery, prostate volume

## Abstract

**Background:**

Holmium/thulium laser enucleation of the prostate (LEP) is an established, size-independent surgical option for benign prostatic hyperplasia, while Aquablation is an emerging robotic waterjet therapy. Until recently, the two had never been compared in a randomized head-to-head trial, and the only prior quantitative synthesis relied on an indirect, transurethral resection of the prostate (TURP)-anchored network comparison. We conducted the first pairwise meta-analysis restricted to studies directly comparing Aquablation and LEP, incorporating the first partially randomized head-to-head trial.

**Methods:**

PubMed, Embase, Cochrane Library, and Scopus were searched for studies directly comparing Aquablation with HoLEP or thulium enucleation, following PRISMA 2020 and AMSTAR 2. The complete search strings for each database, the date of the final search, language and date restrictions, and the duplicate removal process. Outcomes were pooled using random-effects (DerSimonian–Laird) models as mean differences (MD) for continuous and risk ratios (RR) for dichotomous variables; outcomes exceeding *I*^2^ of 95% were reported narratively rather than pooled. Prespecified subgroup analyses stratified studies by prostate volume, and a large propensity-matched claims database reporting 30- and 90-day safety outcomes was analyzed separately. Sensitivity analyses restricted to prospective studies and excluding higher-risk studies were performed to examine robustness.

**Results:**

Of 126 records identified, 7 full-text articles were assessed for eligibility and 1 (ATHLETE) was excluded as a study protocol with no outcome data, leaving 6 studies: 1 partially randomized trial, 4 comparative cohorts (*n* = 845 total), and 1 claims database (2,104 matched patients). Overall symptom relief did not differ significantly [International Prostate Symptom Score (IPSS) MD −0.93, 95% CI −2.13 to 0.27], though Aquablation showed a non-significant numerical trend in large glands (MD −1.21, 95% CI −2.51 to 0.08; exploratory subgroup, k = 2). Laser enucleation provided superior deobstruction, with higher maximum flow rate (Qmax MD −5.66 mL/s, −7.64 to −3.69), lower post-void residual (MD +11.96 mL), and greater tissue removal (residual prostate volume MD +23.4 mL). Aquablation markedly reduced ejaculatory dysfunction (RR 0.19, 0.11–0.32), an effect preserved across gland sizes. Operative time and hemoglobin drop showed extreme heterogeneity and were not pooled. Aquablation was associated with higher 30- and 90-day hospital readmission and catheter reinsertion rates in the claims database, but lower urinary tract infection at 90 days.

**Conclusions:**

No statistically significant difference in overall symptom relief was observed between Aquablation and laser enucleation, though a non-significant numerical trend in large glands is exploratory and requires confirmation. Aquablation confers markedly better, size-independent ejaculatory preservation, while laser enucleation provides superior objective deobstruction. Certainty of evidence remains low to moderate given the limited partially randomized base and short follow-up. These findings support an individualized choice between procedures based on gland size and patient priorities.

**Systematic Review Registration:**

https://www.crd.york.ac.uk/PROSPERO/display_record.php?ID=CRD420261439112, PROSPERO CRD420261439112.

## Introduction

Benign prostatic hyperplasia (BPH) is among the most common causes of bother and lower urinary tract symptoms (LUTS) in aging men, and surgical therapy is indicated when medical management fails. Laser enucleation of the prostate (LEP)—most commonly holmium laser enucleation (HoLEP)—is an anatomical, size-independent technique recommended as a first-line option for larger glands, with durable long-term outcomes (1, 2).

Aquablation (robotic waterjet ablation) is a heat-free, image-guided technique that resects prostatic tissue under transrectal ultrasound mapping while sparing the verumontanum and ejaculatory ducts during contour planning. Randomized data against transurethral resection of the prostate (WATER) and single-arm data in large glands (WATER II) established Aquablation's efficacy and a favorable ejaculatory profile (3, 4). However, until recently no randomized trial had directly compared Aquablation with laser enucleation, and the only quantitative synthesis was an indirect network meta-analysis that bridged the two procedures through a common TURP comparator and therefore depended on the transitivity assumption (5).

The recent publication of the first partially randomized head-to-head trial, together with several comparative cohorts and a large real-world claims database, now permits a direct comparison. We therefore performed a systematic review and pairwise meta-analysis of studies directly comparing Aquablation and laser enucleation, quantifying symptom relief, deobstructive efficacy, ejaculatory preservation, and perioperative safety, while examining whether relative performance varies with prostate size.

## Materials and methods

### Protocol and registration

This systematic review and meta-analysis was structured in accordance with PRISMA 2020 and PRISMA-P guidelines (6), and appraised against AMSTAR 2 (7). The protocol was registered in PROSPERO (CRD420261439112).

### Eligibility criteria

Studies were eligible if they directly compared Aquablation with HoLEP or thulium laser enucleation in adult men with LUTS due to BPH, and reported at least one functional, perioperative, or safety outcome. Randomized trials, propensity-matched series, and prospective or retrospective comparative cohorts were included. Single-arm studies, protocols without outcome data, reviews, and conference abstracts without extractable data were excluded.

### Search strategy and study selection

PubMed, Embase, the Cochrane Library, and Scopus were searched, combining terms for Aquablation (“aquablation”, “waterjet”, “AQUABEAM”) and laser enucleation (“HoLEP”, “thulium enucleation”, “laser enucleation”), supplemented by citation and reference list tracking. The complete search strings for each database, the date of the final search, language and date restrictions, and the duplicate removal process are provided in Supplementary Appendix A. The search was last performed on 15 June 2026. No language restrictions were applied; records published from database inception were considered eligible. Duplicate records were identified using EndNote's automated deduplication tool and manually verified by both reviewers. This search identified 126 records. Following title/abstract screening, seven full-text articles were assessed for eligibility; one (the ATHLETE trial protocol) was excluded because it reported a study protocol with no outcome data, leaving six studies for synthesis (Figure 1).

**Figure 1:**
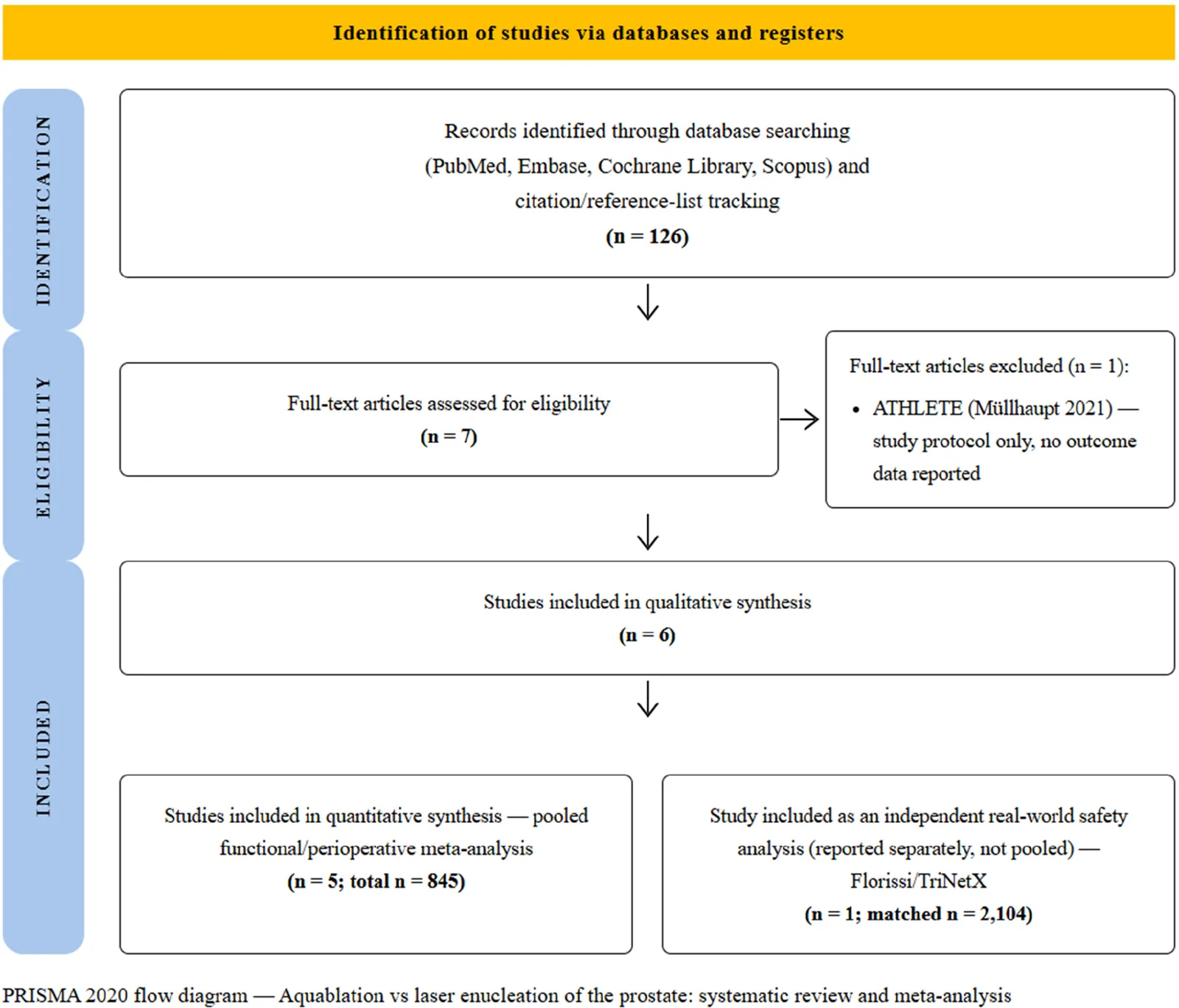
PRISMA 2020 flow diagram of study identification, screening, and inclusion.

### Data extraction and risk of bias

Two reviewers independently extracted study characteristics, baseline parameters, and outcomes across three domains: functional efficacy [IPSS, Qmax, post-void residual volume (PVR), prostate volume], perioperative safety [Clavien–Dindo complications (15), hemoglobin drop, transfusion, and, for the claims database, 30-/90-day readmission, emergency department (ED) visits, urinary tract infection (UTI), and catheter reinsertion], and sexual function [Male Sexual Health Questionnaire for Ejaculatory Dysfunction (MSHQ-EjD), erectile function domain of the International Index of Erectile Function (EF-IIEF), and dichotomous ejaculatory dysfunction]. Risk of bias was assessed using RoB 2 for the randomized trial and the Newcastle–Ottawa Scale (NOS) for observational studies.

### Statistical analysis

Outcomes were pooled using random-effects (DerSimonian–Laird) models (8). Continuous outcomes were expressed as mean differences (MD; Aquablation minus laser enucleation), and dichotomous outcomes as risk ratios (RR; Aquablation versus laser enucleation), with a 0.5 continuity correction for zero cells. Heterogeneity was assessed with *I*^2^; as a prespecified rule, outcomes exceeding *I*^2^ = 95% were considered too heterogeneous to summarize with a single pooled estimate and were instead reported narratively per study. A prespecified subgroup analysis stratified studies by mean prostate volume (medium, <80 mL; large, ≥80 mL). The Florissi/TriNetX claims database was analyzed and reported separately from the pooled functional outcome estimates (14), given its distinct design (administrative claims data, no functional outcomes, different matching covariates). Certainty of evidence was graded using GRADE. Analyses were performed in Python 3 (NumPy/SciPy).

To examine the influence of study quality on pooled estimates, we conducted sensitivity analyses excluding studies at higher risk of bias. For the partially randomized trial, we used RoB 2 domain judgments; for observational studies, we used the NOS, defining higher risk as a score <6/9 or any domain rated as high risk of bias. Additional sensitivity analyses comprised the following: (1) a prospective-only analysis excluding the retrospective propensity-score-matched cohort (12); (2) exclusion of the highest-risk observational study (13) (NOS 5/9); and (3) reporting of design-stratified estimates to examine robustness to internal validity heterogeneity.

## Results

### Study selection and characteristics

Five comparative studies met inclusion criteria for functional/perioperative pooling, comprising 845 patients (9–13). One was a partially randomized non-inferiority trial (WATER III) comparing Aquablation with laser enucleation (HoLEP or thulium) in large glands (80–180 mL). A second was a prospective study directly comparing Aquablation with HoLEP and TURP in medium to large glands (Perez et al.). The remaining three were comparative studies of Aquablation versus HoLEP in medium-sized glands. A sixth study, a large propensity-matched claims database (TriNetX; 1,052 Aquablation vs. 1,052 HoLEP after matching), contributed independent real-world 30- and 90-day safety data (14). Baseline characteristics are summarized in Table 1.

**Table 1:** Characteristics of included studies.

Study	Design	*n* (Aqua/LEP)	Mean PV (mL)	Size band	Follow-up
WATER III (9)	Partially randomized RCT	98/88	∼106 (80–180)	Large	3 months (5-year planned)
Perez et al. (10)	Prospective vs. retrospective	318/83^a^	94 vs. 105	Large	3/6/12/24 months
Justo Quintas et al. (11)	Prospective comparative	75/75	∼72–82	Medium	6 months
Oh and Kim (12)	Retrospective PSM	34/34	∼70	Medium	∼10 months
Michaelis et al. (13)	Prospective non-randomized	16/24	55–74	Medium	3/6/12 months
Florissi et al. (14) (TriNetX)^b^	Retrospective PSM claims DB	1,052/1,052	not captured	–	30/90 days

PV, prostate volume; PSM, propensity score matching.

a Perez et al. also included a TURP arm (*n* = 83), not used in this HoLEP-focused comparison.

b Florissi/TriNetX contributed safety outcomes only and was not pooled with the functional outcome studies.

### Symptom relief (IPSS)

Overall symptom relief did not differ significantly between procedures (IPSS MD −0.93, 95% CI −2.13 to 0.27; *p* = 0.128; *I*^2^ = 38%; Figure 2). The WATER III trial formally established non-inferiority of Aquablation for IPSS improvement at 3 months. Perez et al. reported a numerically greater IPSS reduction with Aquablation (17.4-point drop) than HoLEP (14.7-point drop). In the size-stratified exploratory subgroup, Aquablation showed a non-significant numerical trend in large glands (MD −1.21, 95% CI −2.51 to 0.08; *I*^2^ = 0%; k = 2; Figure 3). Because the CI crossed zero and only two studies contributed, this exploratory finding should not be interpreted as evidence of superiority. In medium glands, the difference was also non-significant (MD −0.59, −2.91 to 1.74; *I*^2^ = 65%).

**Figure 2:**
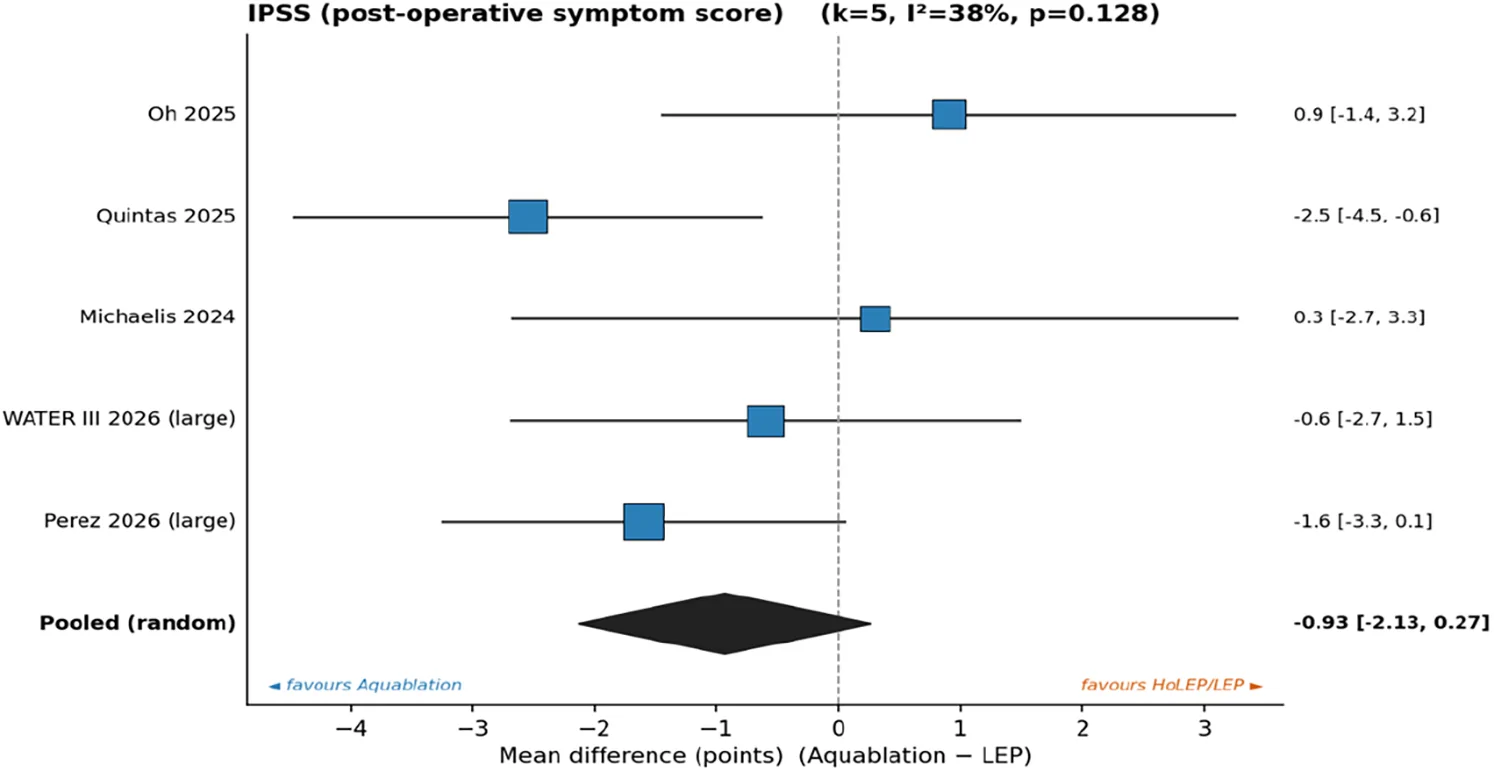
Forest plot, IPSS (postoperative symptom score). Negative mean differences favor Aquablation.

**Figure 3:**
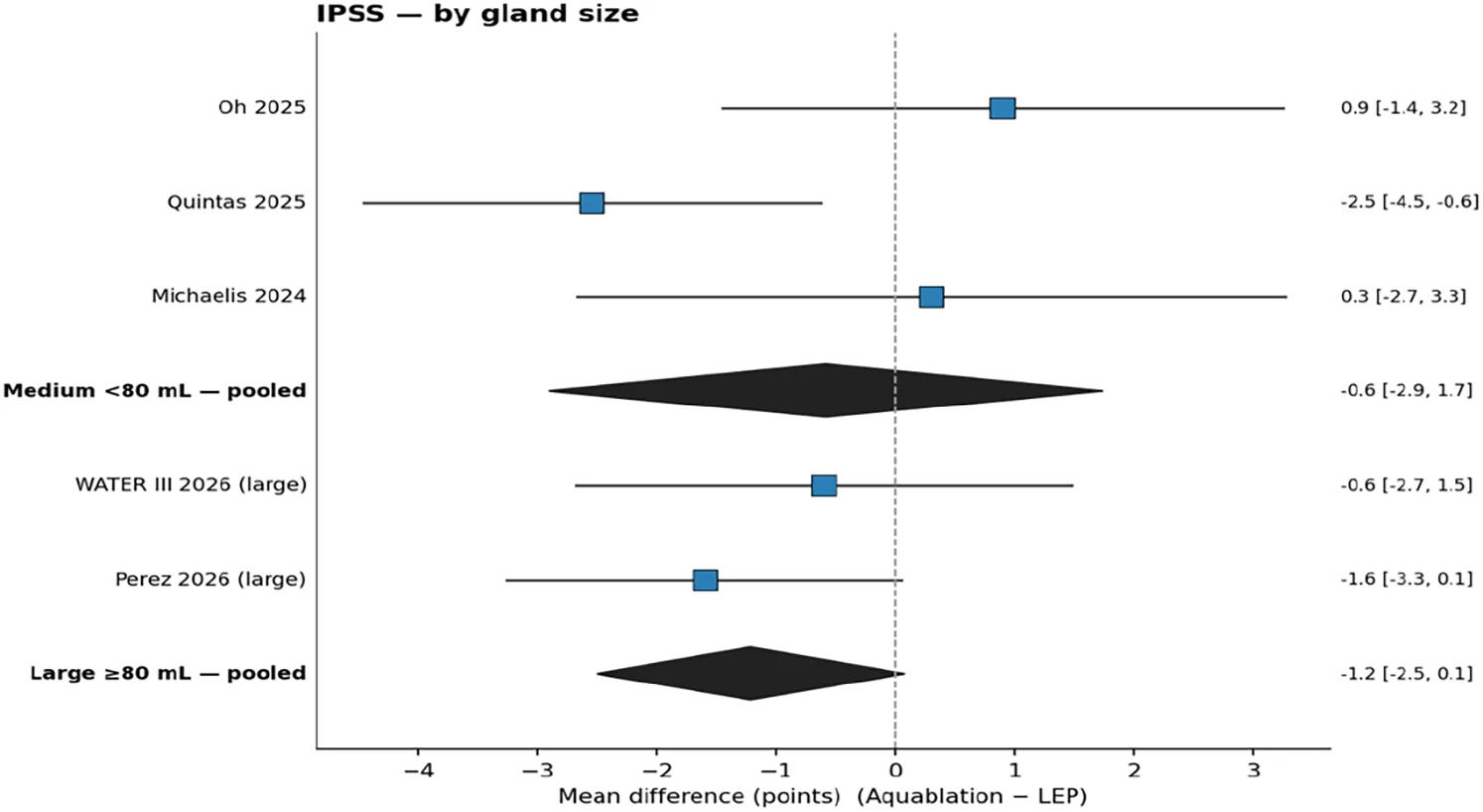
IPSS stratified by prostate size band (medium <80 mL vs. large ≥80 mL). The large-gland estimate (k = 2) is exploratory and the 95% CI crosses zero, indicating a non-significant directional trend.

### Deobstructive efficacy (Qmax, PVR, tissue removal)

Data consistently favored laser enucleation for objective deobstruction. Pooled across four studies, Qmax favored LEP by MD −5.66 mL/s (−7.64 to −3.69; *I*^2^ = 16%; Figure 4). Post-void residual was likewise lower after LEP (pooled MD +11.96 mL favoring LEP, 7.26–16.67; *I*^2^ = 0%; Figure 5), and tissue removal was substantially greater after LEP (residual prostate volume MD +23.4 mL favoring LEP, 13.3–33.5; k = 2; Figure 6). Pooled estimates are summarized in Table 2.

**Figure 4:**
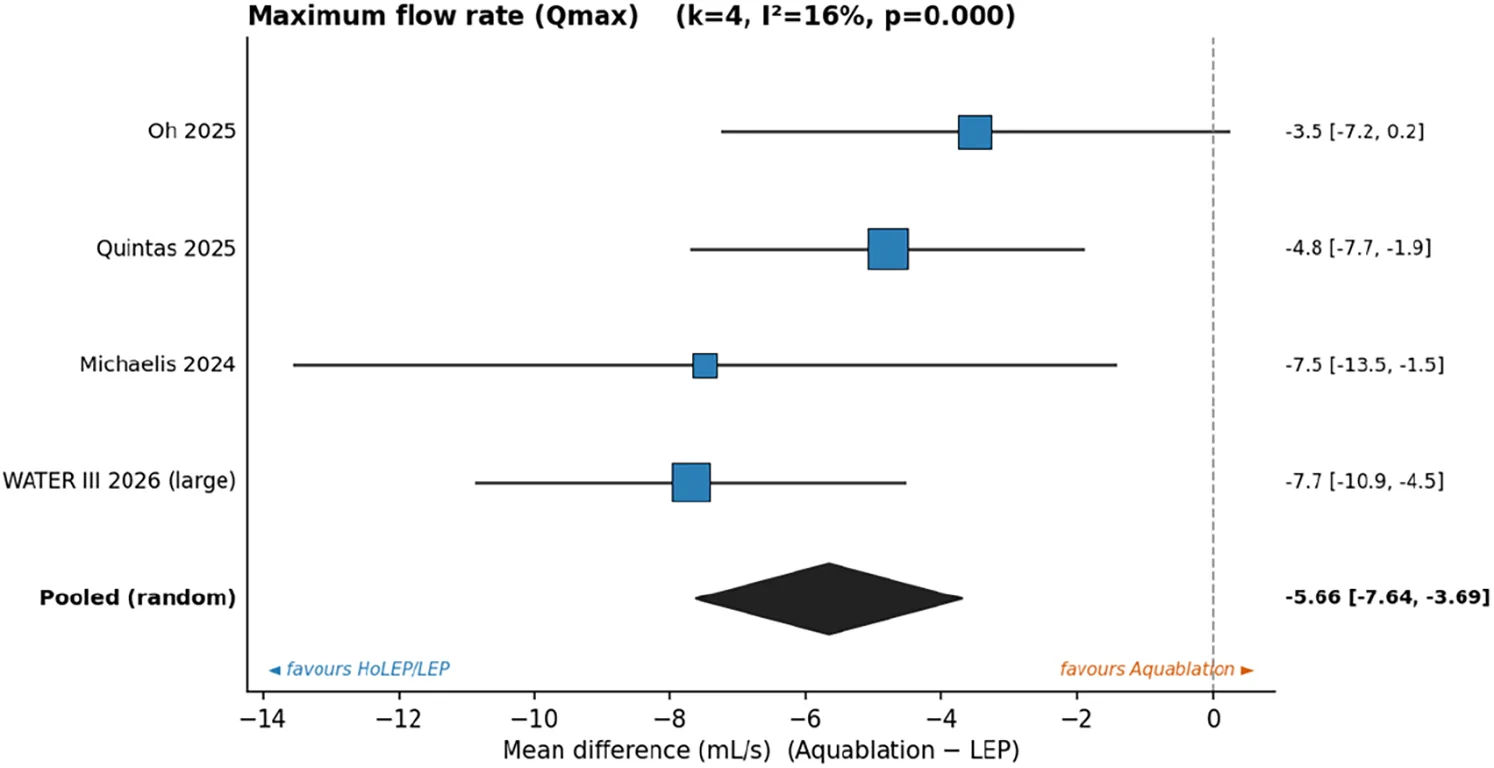
Forest plot, maximum urinary flow rate (Qmax). Negative mean differences favor laser enucleation.

**Figure 5:**
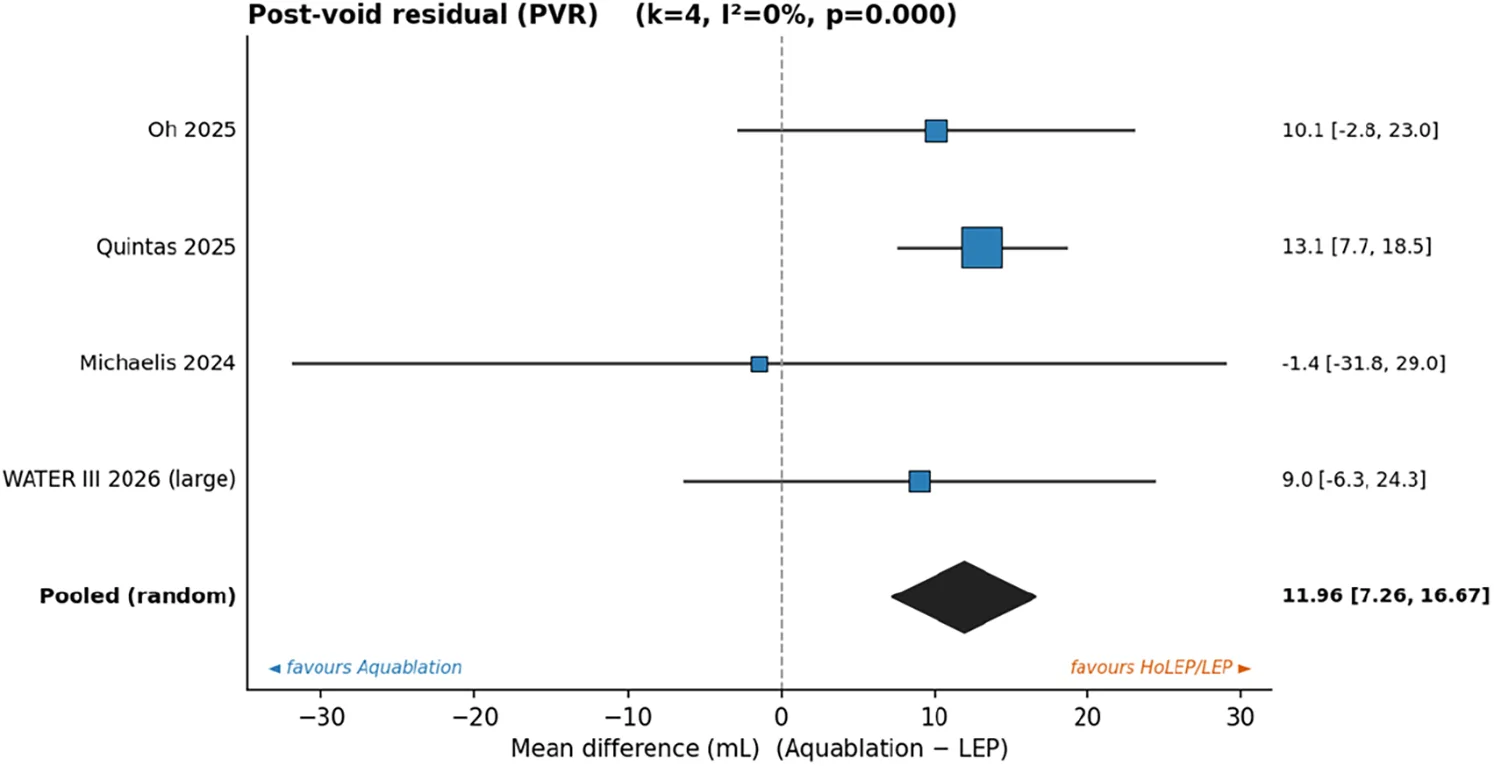
Forest plot, post-void residual volume (PVR).

**Figure 6:**
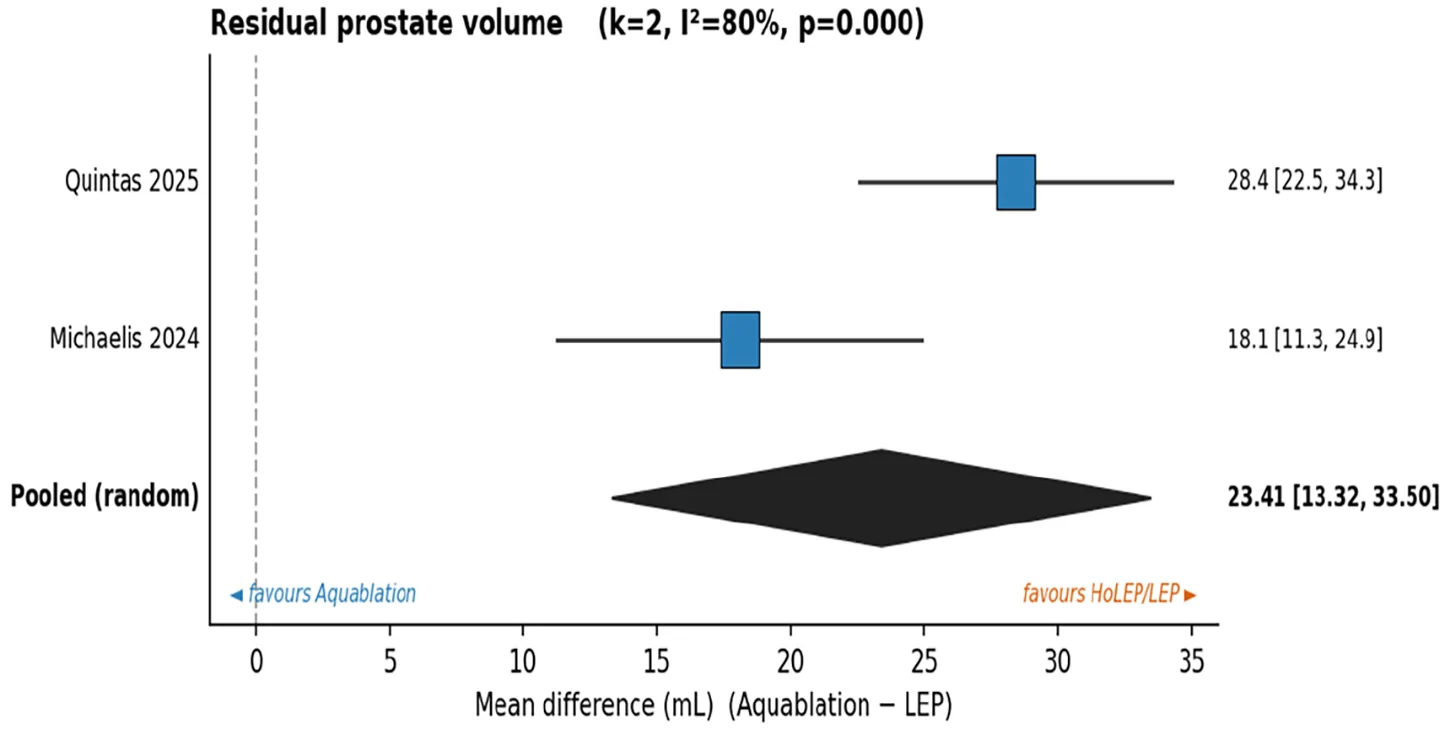
Forest plot, residual prostate volume (tissue removed).

**Table 2:** Pooled random-effects estimates.

Outcome	k	Estimate (95% CI)	*p*	*I*^2^ (%)
IPSS (post-op)	5	MD −0.93 (−2.13 to 0.27)	0.128	38
Qmax (mL/s)	4	MD −5.66 (−7.64 to −3.69)	<0.001	16
PVR (mL)	4	MD +11.96 (7.26 to 16.67)	<0.001	0
Residual prostate volume (mL)	2	MD +23.41 (13.32 to 33.50)	<0.001	80
Hospital stay (days)	4	MD +0.08 (−0.05 to 0.22)	0.222	0
Blood transfusion	4	RR 0.55 (0.11 to 2.63)	0.451	0
Ejaculatory dysfunction	4	RR 0.19 (0.11 to 0.32)	<0.001	61

MD, mean difference (Aquablation − laser enucleation); RR, risk ratio (Aquablation vs. laser enucleation); k, number of studies.

### Outcomes not pooled (extreme heterogeneity)

Total operative time (k = 5) and hemoglobin drop (k = 2) showed extreme, uninterpretable heterogeneity (*I*^2^ = 98% and 99%, respectively) and were reported narratively rather than as a single pooled estimate (Table 3; Figure 7).

**Table 3:** Outcomes with *I*^2^ > 95%, reported narratively per prespecified rule rather than pooled.

Outcome	Study	Aquablation	LEP
Operative time (min)	Oh and Kim (12)	87.6 (*n* = 34)	99.6 (*n* = 34)
	Justo Quintas et al. (11)	49.1 (*n* = 75)	43.9 (*n* = 75)
	Michaelis et al. (13)	87.2 (*n* = 16)	59.5 (*n* = 24)
	WATER III (9)	58.1 (*n* = 98)	61.8 (*n* = 88)
	Perez et al. (10)	39.0 (*n* = 318)	91.2 (*n* = 83)
Hemoglobin drop (g/dL)	Quintas et al. (11)	2.6 (*n* = 75)	0.4 (*n* = 75)
	Perez et al. (10)	2.4 (*n* = 318)	2.1 (*n* = 83)

**Figure 7:**
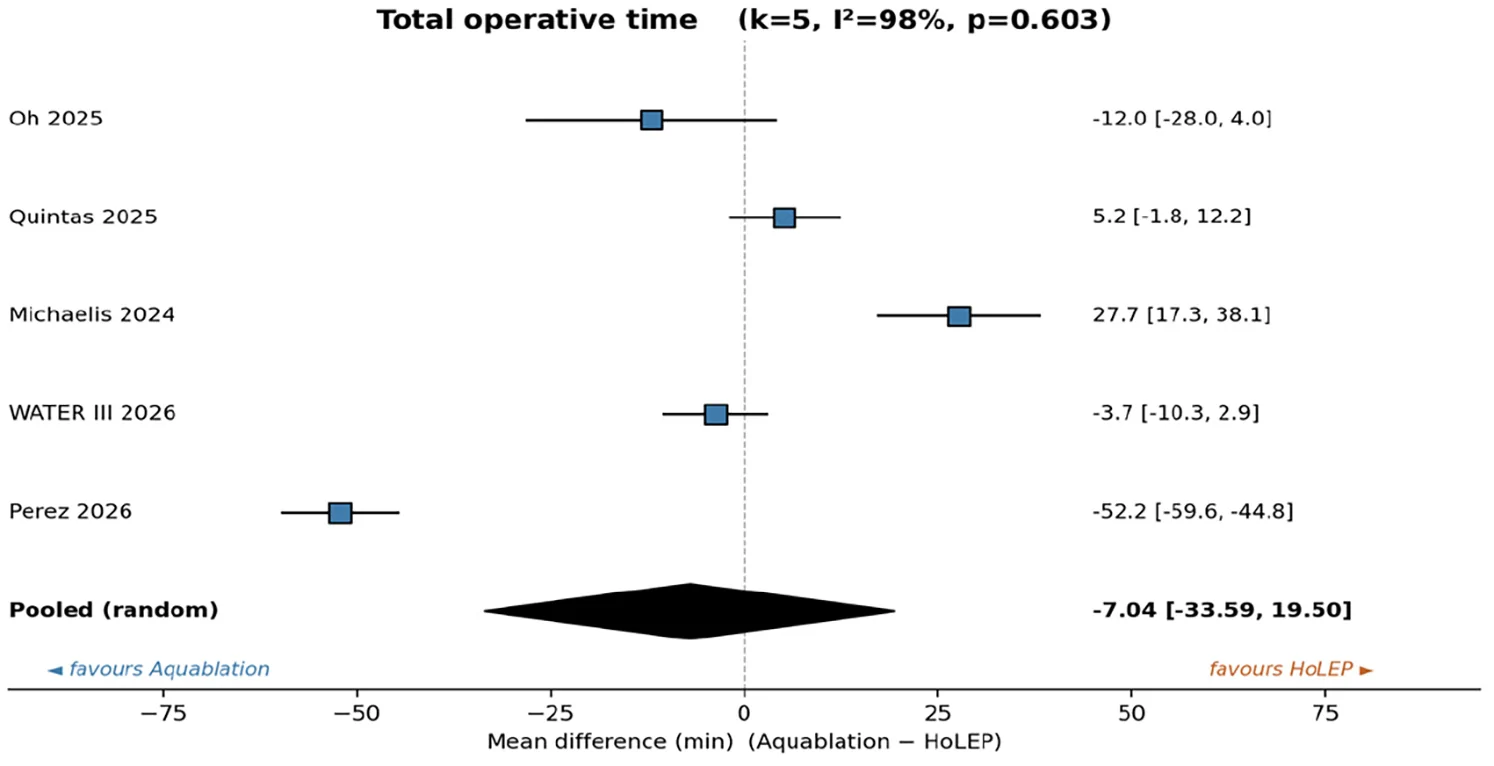
Operative time by study, including Perez et al. (10); shown for completeness; NOT pooled (k = 5, I2 = 98%, see Table 3).

### Sexual function

Aquablation was associated with a markedly lower risk of ejaculatory dysfunction (RR 0.19, 0.11–0.32; k = 4; Figure 8), preserved across gland-size bands (medium RR 0.17; large RR 0.20). Blood transfusion was rare and did not differ (RR 0.55, 0.11–2.63; k = 4, including Gloger et al. (16); Figure 9). Hospital stay did not differ significantly (Figure 10). As this analysis was not powered or designed as a non-inferiority study, these findings should not be read as evidence of equivalence.

**Figure 8:**
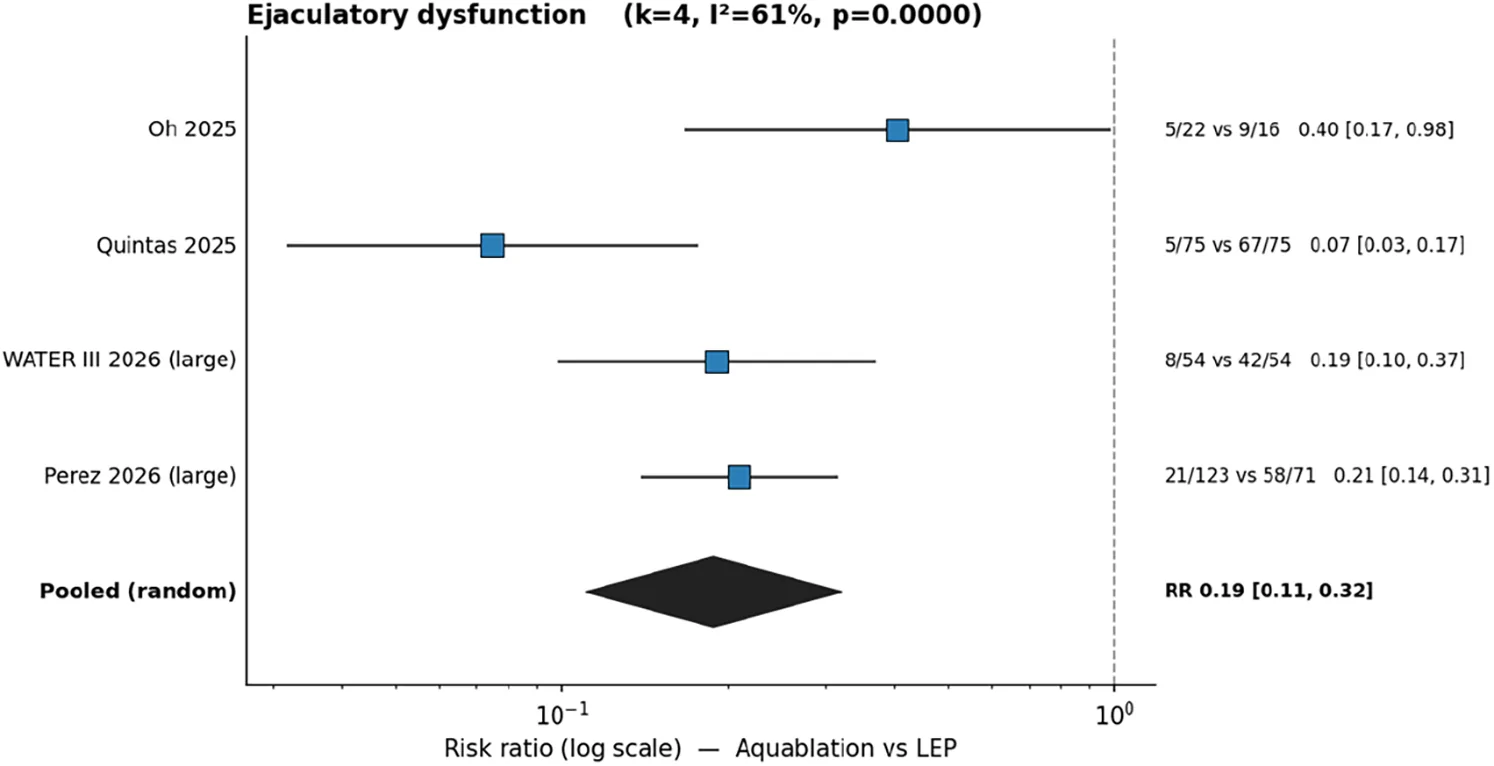
Forest plot, ejaculatory dysfunction (risk ratio, Aquablation vs. laser enucleation).

**Figure 9:**
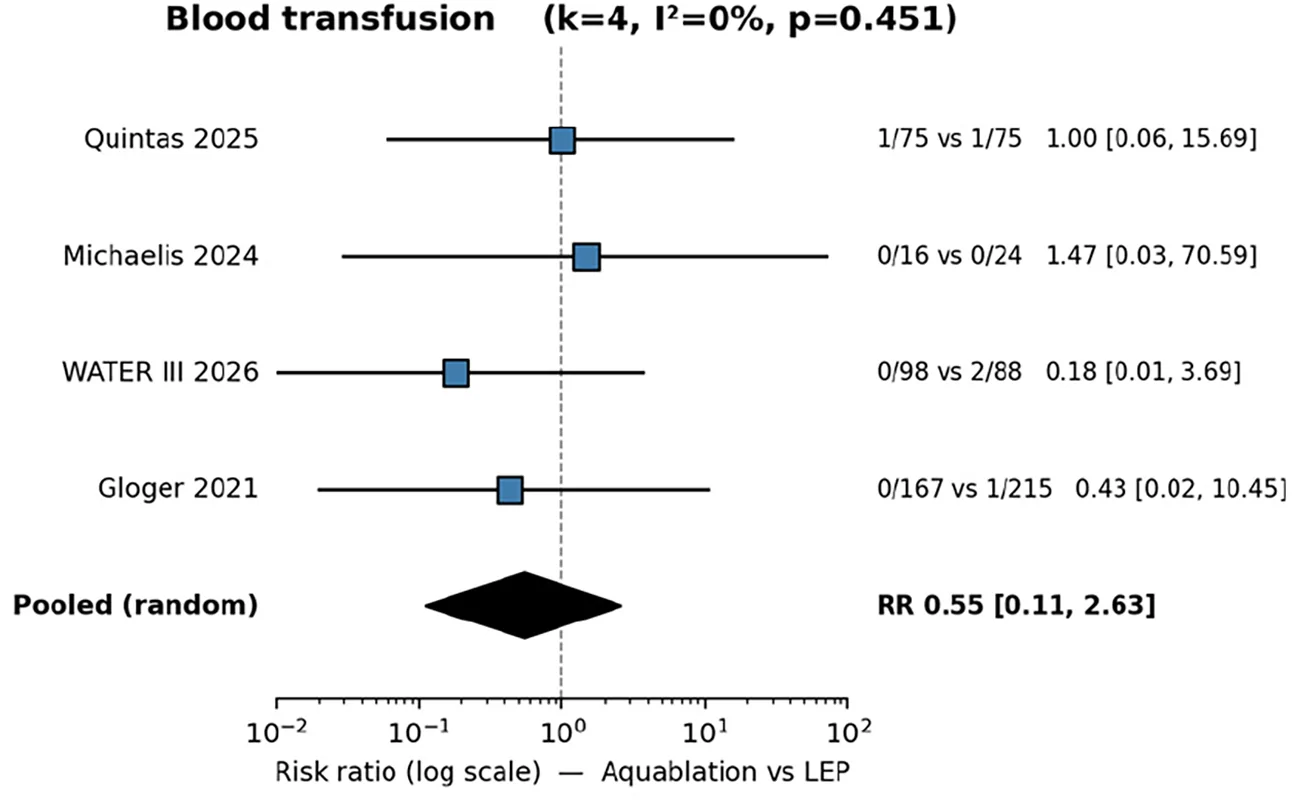
Forest plot, blood transfusion (risk ratio, Aquablation vs. laser enucleation).

**Figure 10:**
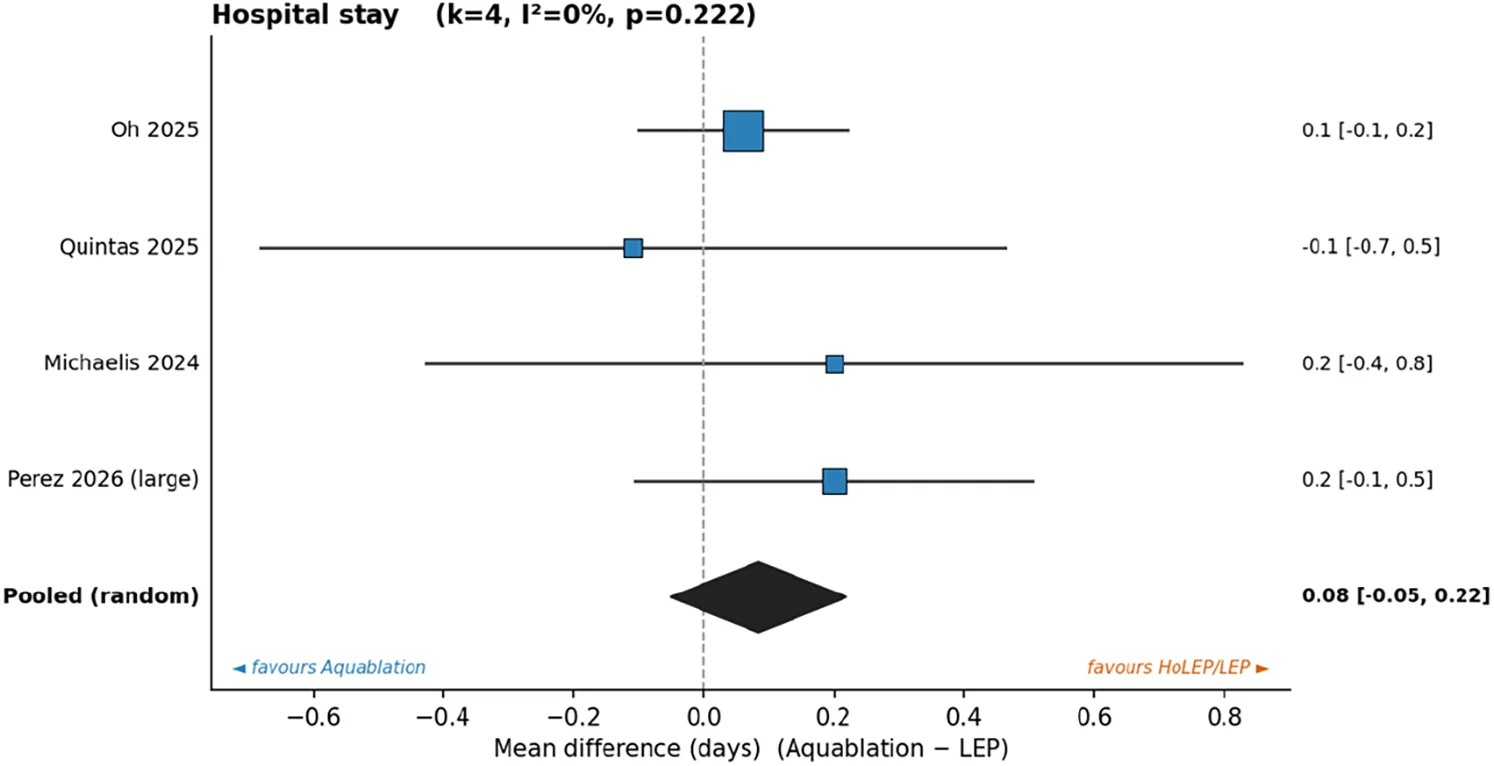
Forest plot, hospital stay (days).

### Real-world safety [Florissi et al., TriNetX]

In the propensity-matched claims database, Aquablation was associated with significantly higher 30-day hospital readmission (RR 4.84, 3.32–7.06) and catheter reinsertion (RR 2.35, 1.40–3.94), persisting at 90 days (readmission RR 4.22, 2.98–5.97; catheter reinsertion RR 2.46, 1.51–4.00), but lower urinary tract infection at 90 days (RR 0.69, 0.52–0.92) (14). The source publication reported that this readmission signal was concentrated in the year of steepest Aquablation case volume growth and did not persist after excluding those cases. As an administrative claims database, this cannot establish a causal learning curve mechanism, and we interpret this temporal pattern as hypothesis-generating only (Table 4; Figure 11).

**Table 4:** Florissi et al. (14) (TriNetX)—matched real-world safety outcomes, Aquablation vs. HoLEP.

Outcome	30-day RR (95% CI)	90-day RR (95% CI)
Hospital readmission	4.84 (3.32–7.06)	4.22 (2.98–5.97)
Catheter reinsertion	2.35 (1.40–3.94)	2.46 (1.51–4.00)
Urinary tract infection	0.80 (0.56–1.14)	0.69 (0.52–0.92)
Emergency department visit	1.18 (0.85–1.63)	1.02 (0.78–1.34)

RR > 1 indicates higher risk with Aquablation.

**Figure 11:**
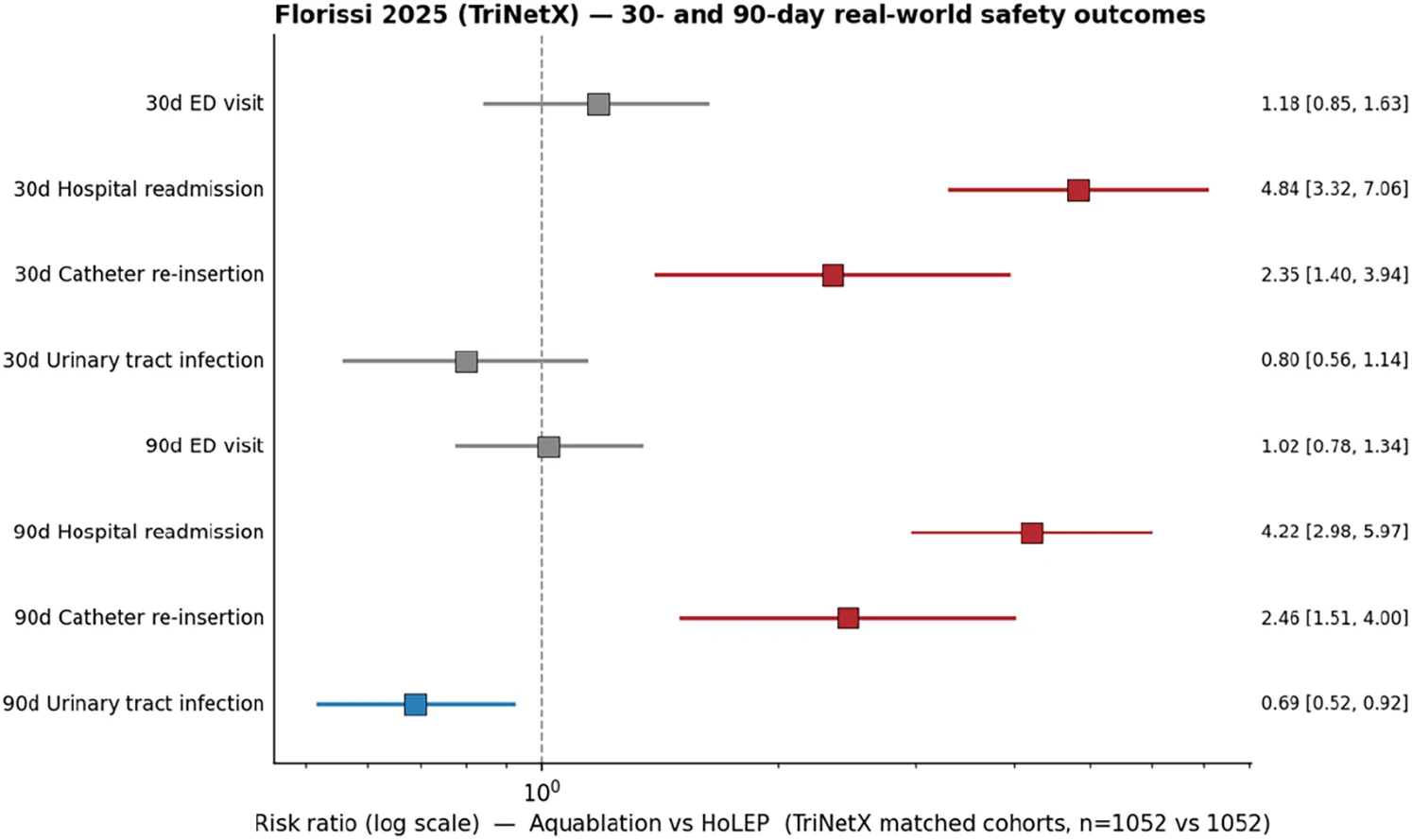
Florissi et al. (14) (TriNetX)—30- and 90-day real-world safety outcomes, Aquablation vs. HoLEP (matched cohorts, *n* = 1,052 vs. 1,052).

### Subgroup analysis by prostate size

This size-stratified analysis is exploratory: Stratification by study-level mean prostate volume is not equivalent to a patient-level treatment-by-volume interaction, and the large-gland subgroup contains only two studies. Therefore, it cannot establish effect modification and should be interpreted with caution.

The large-gland band comprised two studies (WATER III and Perez et al.). Symptom relief showed a non-significant numerical trend favoring Aquablation in the exploratory large-gland subgroup (Figure 3). The Qmax deobstruction advantage for LEP widened in large glands (Figure 12). Residual prostate volume by size band is shown in Figure 13. Aquablation's ejaculatory advantage was preserved in both bands (Table 5; Figure 7).

**Figure 12:**
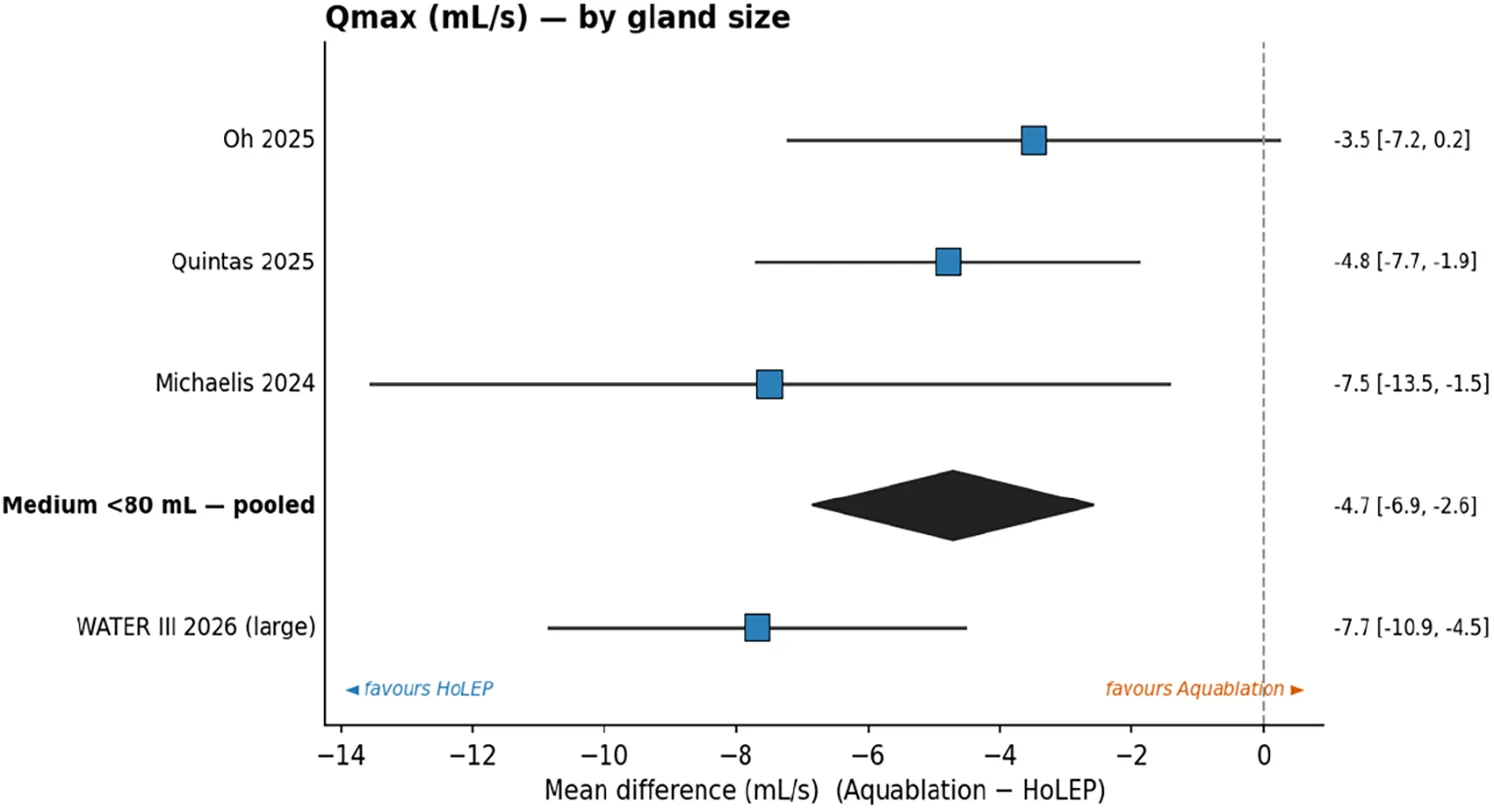
Qmax stratified by prostate size band; laser enucleation's flow advantage widens in large glands.

**Figure 13:**
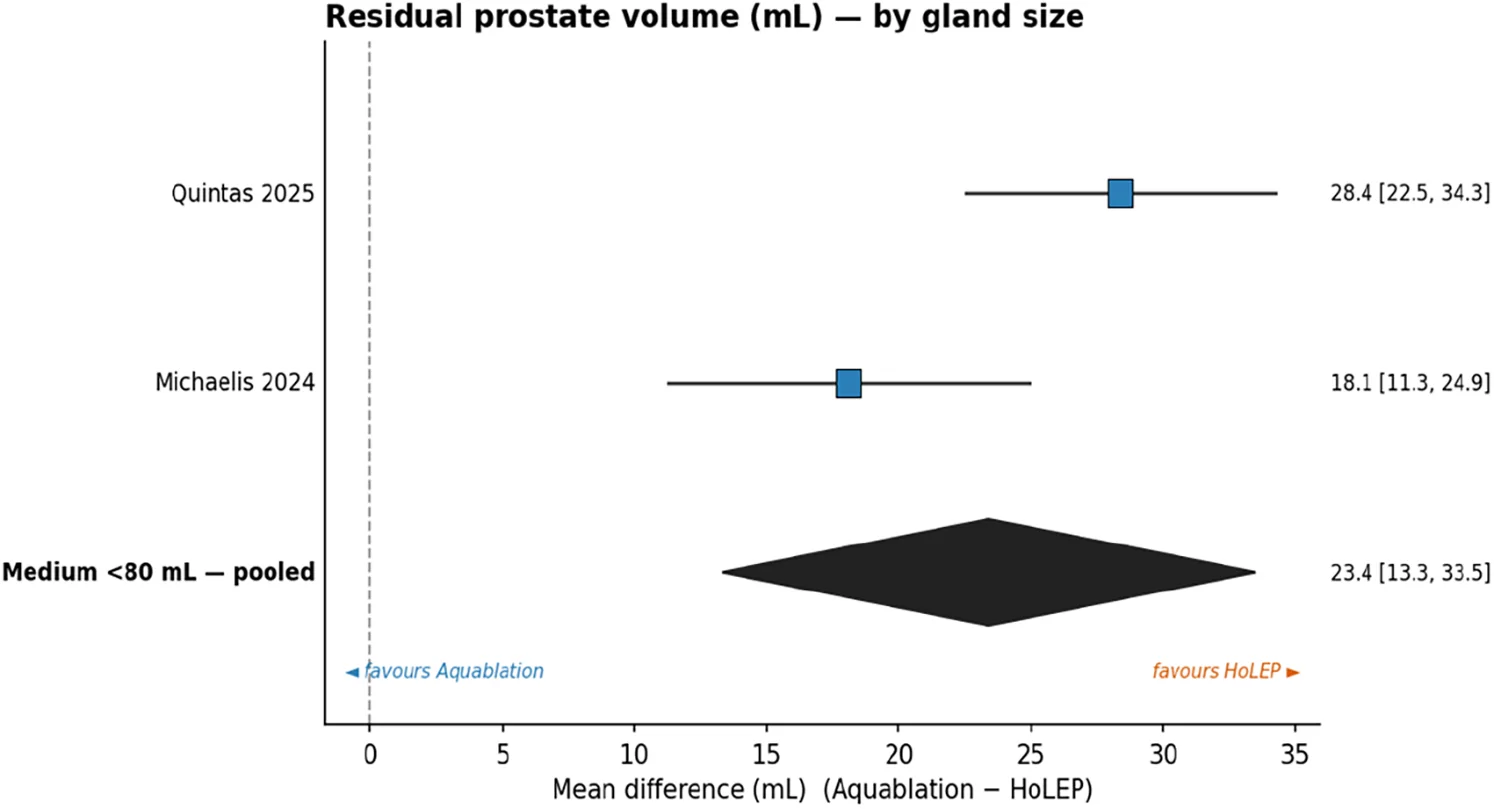
Residual prostate volume stratified by prostate size band.

**Table 5:** Subgroup analyses by prostate size.

Outcome	Size band	k	Estimate	95% CI	*I*^2^ (%)
IPSS	Medium (<80 mL)	3	−0.59	−2.91 to 1.74	65
IPSS	Large (≥80 mL)	2	−1.21	−2.51 to 0.08	0
Ejaculatory dysfunction (RR)	Medium (<80 mL)	2	0.17	0.03 to 0.90	—
Ejaculatory dysfunction (RR)	Large (≥80 mL)	2	0.20	0.14 to 0.29	—

Large-gland band comprises WATER III and Perez (k = 2); findings in this band are exploratory and should be interpreted with caution given the limited evidence base. Medium-gland band comprises Oh, Quintas, and Michaelis.

### Sensitivity analyses

Sensitivity analyses stratified by study design are presented in Supplementary Table S1. Because only one study was randomized (WATER III, and only partially), a randomized-only stratum could not be pooled; design-stratified analysis was therefore operationalized as exclusion of the retrospective propensity-score-matched cohort (12). For IPSS, this yielded MD −1.39 (95% CI −2.45 to −0.33; *I*^2^ = 8%; k = 4). The Qmax advantage for laser enucleation and the ejaculatory preservation advantage for Aquablation persisted across all design strata. Sensitivity analyses excluding the highest-risk observational study (13) (NOS 5/9) are presented in Supplementary Table S2 and did not reverse the direction of any effect: IPSS MD −1.07 (−2.41 to 0.28; *I*^2^ = 45%; k = 4), Qmax MD −5.42 (−7.77 to −3.06; *I*^2^ = 37%; k = 3), PVR MD +12.30 (7.57–17.04; *I*^2^ = 0%; k = 3), and hospital stay MD +0.11 (−0.02 to 0.24; *I*^2^ = 0%; k = 3). Michaelis et al. (13) did not contribute to the ejaculatory dysfunction outcome, so that estimate is unchanged by this exclusion. Across both sensitivity frameworks, the direction of every effect was preserved; statistical significance for IPSS was sensitive to which study was removed, reinforcing the idea that no symptom relief advantage should be claimed for either technique. Risk-of-bias assessments with domain-level justifications are summarized in Supplementary Figure S1. Using RoB 2, the partially randomized trial (WATER III) was judged to have some concerns overall, driven by the randomization process (combined randomized and non-randomized cohorts via Bayesian borrowing), deviations from intended interventions (open-label design), and measurement of the outcome (unblinded patient-reported IPSS). The missing data and result selection domains were rated low risk. Among the five observational studies (Newcastle–Ottawa Scale), scores ranged from 3 to 8 (median 5): Justo Quintas et al. (11) (8/9, low risk), Florissi et al. (14)/TriNetX (6/9, some concerns), Oh and Kim (12) and Michaelis et al. (13) (5/9 each, some concerns), and Perez et al. (10) (3/9, high risk, reflecting its retrospective non-concurrent comparator, lack of confounder adjustment, and approximately 30% retention at 12 months).

## Discussion

In this pairwise meta-analysis restricted to head-to-head studies and incorporating the first partially randomized trial, Aquablation and laser enucleation showed no statistically significant difference in overall symptom relief or length of stay. Because this review was not designed as a non-inferiority study and no equivalence margin was prespecified, these non-significant differences indicate an absence of demonstrated difference rather than demonstrated equivalence. The two procedures diverged along two consistent axes: Laser enucleation delivered superior objective deobstruction, whereas Aquablation conferred a substantial and size-independent advantage in ejaculatory preservation. A large real-world claims database added an important counterweight: Aquablation was associated with a materially higher risk of early hospital readmission and catheter reinsertion, a pattern concentrated in the period of most rapid case volume growth. Because these are administrative claims data, this temporal pattern is an association only and cannot establish a learning curve mechanism.

Our findings both confirm and extend the previous indirect network meta-analysis, which—constrained by the absence of any direct comparison at its search date—could only infer the relationship between the two procedures through a TURP anchor (5). By restricting to direct, within-study comparisons, including the first partially randomized head-to-head trial, and incorporating a second, larger prospective comparison in large glands, the present analysis provides more direct comparative evidence without reliance on the transitivity assumption of the previous network meta-analysis.

The size-stratified findings carry practical weight, but must be interpreted cautiously. The large-gland subgroup is based on only two studies (one partially randomized trial and one prospective comparative study), and the IPSS estimate for this subgroup, while showing a non-significant numerical trend favoring Aquablation, did not achieve statistical significance (95% CI −2.51 to 0.08). We therefore describe these subgroup findings as exploratory and emphasize that they require confirmation in larger, dedicated trials in very large prostates. Stratification by study-level mean prostate volume is not equivalent to a patient-level treatment-by-volume interaction; therefore, this analysis cannot establish effect modification. Even with a second large-gland study now available, symptom relief did not differ significantly between procedures in this exploratory large-gland subgroup, whereas Aquablation's ejaculatory advantage was preserved regardless of gland size. The real-world safety data deserve particular emphasis because they derive from a source structurally different from, and less prone to enthusiasm bias than, investigator-led comparative cohorts. The concentration of the readmission signal in the highest-volume year, and its attenuation after excluding that year, is compatible with a learning curve effect. However, an administrative claims database cannot establish this or any other causal mechanism: Coding practices, case-mix, and institutional adoption patterns are unmeasured and confounded with calendar time. This temporal association should therefore be regarded as hypothesis-generating and kept clearly distinct from the comparative clinical studies pooled previously, which alone support inference about relative treatment effect.

The extreme heterogeneity in operative time warrants explicit consideration of its sources, since study-level estimates conflict in both direction and magnitude. At least four factors plausibly contribute. First, prostate volume differed substantially across cohorts (mean ∼55–106 mL), and the two techniques scale differently with gland size: Aquablation resection time is largely volume-independent because the waterjet phase is planned and automated, whereas enucleation and particularly morcellation time increase with adenoma volume. Studies enriched for larger glands will therefore favor Aquablation on time, and those in medium glands will not. Second, surgeon experience is unevenly distributed: Laser enucleation has a long and well-documented learning curve, and the comparator arms in these studies were generally performed by established high-volume enucleators, whereas the Aquablation arms often represented early institutional experience. Third, the laser platform and enucleation technique were not uniform—holmium in four studies and holmium or thulium in one—with differing enucleation strategies and morcellator performance, all of which materially affect operative duration. Fourth, and most importantly for interpretation, the definition of “operative time” was not standardized: Some reports capture console or resection time only, whereas others include cystoscopy, planning, mapping, hemostasis, and morcellation. This last source of variation is a reporting artifact rather than a true clinical difference and on its own is sufficient to render a pooled estimate uninterpretable. For these reasons, operative time is presented narratively rather than as a single summary estimate, and no claim of superiority in operative efficiency is made for either technique.

### Strengths and limitations

Strengths of this review include restriction to direct comparative evidence, inclusion of the first partially randomized head-to-head trial, addition of a second large-gland comparative study, use of a prespecified size subgroup, incorporation of a large independent real-world safety dataset, and application of a prespecified rule preventing the pooling of outcomes with uninterpretable heterogeneity. Several limitations should be noted. Only one study was (partially) randomized, so certainty was rated low to moderate for most outcomes, and few studies contributed to most outcomes (k = 2–5). Follow-up was short (3–24 months): No study reported retreatment rates, time-to-retreatment, or durability of symptom relief beyond 2 years. None of the studies had follow-up sufficient enough to detect late failure, and the longest reported follow-up (24 months) remains well short of the 5-year benchmarks typically used to judge durability in BPH surgery. Long-term comparative durability between the two techniques therefore remains unknown from this evidence base. The comparator was not perfectly uniform (HoLEP in four studies, HoLEP or thulium in one). Operative time and hemoglobin drop could not be meaningfully pooled and are reported narratively. The Florissi/TriNetX safety analysis, while large, is a distinct claims-based data source unable to control for prostate volume. Ejaculatory dysfunction denominators were subgroup-specific and partly approximated, so the pooled risk ratio retains moderate heterogeneity (*I*^2^ = 61%). Sensitivity analyses restricted to prospective studies and excluding the highest-risk observational study yielded directionally consistent results, although statistical significance was sensitive to the inclusion of the retrospective propensity-score-matched study.

## Conclusions

On current direct, partly randomized evidence, no statistically significant difference in overall symptom relief was observed between Aquablation and laser enucleation. A non-significant numerical trend in large glands is exploratory and requires confirmation. Aquablation confers markedly superior, size-independent ejaculatory preservation, whereas laser enucleation provides superior objective deobstruction. A large real-world database indicates a more complex early postoperative course after Aquablation. As this piece of evidence derives from administrative claims data, a learning curve mechanism is plausible but cannot be established; this association should be interpreted as hypothesis-generating. The choice between procedures can be individualized according to prostate size, the relative priority of ejaculatory preservation versus maximal deobstruction, and institutional experience. Larger, randomized, longer-term head-to-head data—particularly flow and durability outcomes in very large glands—are needed to confirm and extend these findings.

## Data Availability

The raw data supporting the conclusions of this article will be made available by the authors without undue reservation.
