# Supplementary material for: Aquablation versus laser enucleation of the prostate for benign prostatic hyperplasia: a systematic review and pairwise meta-analysis of head-to-head studies including the first randomized trial
Source: Front Surg. 2026 Sep 21;13:1937749. doi: 10.3389/fsurg.2026.1937749 (PMC13635371; doi:10.3389/fsurg.2026.1937749)
Supplement: Supplementary file 1 [file Datasheet1.pdf]

Supplementary Material

Aquablation versus Laser Enucleation of the Prostate for Benign Prostatic Hyperplasia: A Systematic Review and Pairwise Meta-Analysis of Head-to-Head Studies Including the First Randomized Trial

Manuscript ID 1937749 — Frontiers in Surgery, Genitourinary Surgery and Interventions

PROSPERO CRD420261439112

Contents:

- Appendix A. Complete reproducible search strategies
- Table S1. Sensitivity analyses stratified by study design
- Table S2. Sensitivity analysis excluding highest-risk study (Michaelis 2024)
- Table S3. GRADE summary of findings
- Figure S1. Risk-of-bias assessment of included studies

Appendix A. Complete Reproducible Search Strategies

Search Date and Parameters

Final search date: 15 June 2026

Language restrictions: None

Date range: Database inception to 15 June 2026

Deduplication: Records were imported into EndNote 21. Automated deduplication was performed using the ‘Find Duplicates’ function with preference set to match on title, author, and year. All suspected duplicates were manually verified by both reviewers before exclusion.

PubMed (via NCBI)

```
("aquablation"[Title/Abstract] OR "waterjet"[Title/Abstract] OR
"AQUABEAM"[Title/Abstract]) AND ("holmium laser enucleation"[Title/Abstract] OR
"HoLEP"[Title/Abstract] OR "thulium enucleation"[Title/Abstract] OR "laser
enucleation"[Title/Abstract])
```

Embase (via Elsevier)

```
('aquablation':ti,ab OR 'waterjet':ti,ab OR 'AQUABEAM':ti,ab) AND ('holmium laser
enucleation':ti,ab OR 'HoLEP':ti,ab OR 'thulium enucleation':ti,ab OR 'laser
enucleation':ti,ab)
```

Cochrane Library (via Wiley)

```
[aquablation OR waterjet OR AQUABEAM] AND [holmium laser enucleation OR HoLEP OR thulium
enucleation OR laser enucleation]
```

Scopus (via Elsevier)

```
(TITLE-ABS("aquablation") OR TITLE-ABS("waterjet") OR TITLE-ABS("AQUABEAM")) AND (TITLE-
ABS("holmium laser enucleation") OR TITLE-ABS("HoLEP") OR TITLE-ABS("thulium
enucleation") OR TITLE-ABS("laser enucleation"))
```

Supplementary Citation and Reference-List Tracking

The reference lists of all included full-text articles and relevant systematic reviews were hand-searched. Forward citation tracking was performed using Google Scholar for all included studies.

Table S1. Sensitivity Analyses Stratified by Study Design

Outcome	Analysis	k	Estimate (95% CI)	I^2 (%)
IPSS (post-op)	Primary (all studies)	5	MD −0.93 (−2.13, 0.27)	38
IPSS (post-op)	Excl. retrospective PSM (Oh & Kim 2025)	4	MD −1.39 (−2.45, −0.33)	8
Qmax (mL/s)	Primary (all studies)	4	MD −5.66 (−7.64, −3.69)	16
Qmax (mL/s)	Excl. retrospective PSM	3	MD −6.27 (−8.29, −4.24)	0
PVR (mL)	Primary (all studies)	4	MD +11.96 (7.26, 16.67)	0
Ejaculatory dysfunction (RR)	Primary (all studies)	4	RR 0.19 (0.11, 0.32)	61
Ejaculatory dysfunction (RR)	Excl. retrospective PSM	3	RR 0.16 (0.09, 0.28)	61
Hospital stay (days)	Primary (all studies)	4	MD +0.08 (−0.05, 0.22)	0

Note: MD, mean difference (Aquablation − laser enucleation); RR, risk ratio (Aquablation vs laser enucleation); k, number of studies; PSM, propensity score matching. All three sensitivity estimates excluding Oh & Kim 2025 (IPSS, Qmax, and ejaculatory dysfunction) were independently recalculated from the original extracted study-level data (DerSimonian–Laird random-effects); the IPSS estimate matches the value reported in the main text and in the response to Reviewer 1, Comment 1.

Table S2. Sensitivity Analysis Excluding Highest-Risk Study (Michaelis 2024; NOS 5/9)

Outcome	k	Primary Estimate	Sensitivity Estimate	I^2 (%)	Direction consistent?
IPSS (post-op)	4	MD −0.93 (−2.13, 0.27)	MD −1.07 (−2.41, 0.28)	45	Yes
Qmax (mL/s)	3	MD −5.66 (−7.64, −3.69)	MD −5.42 (−7.77, −3.06)	37	Yes
PVR (mL)	3	MD +11.96 (7.26, 16.67)	MD +12.30 (7.57, 17.04)	0	Yes
Hospital stay (days)	3	MD +0.08 (−0.05, 0.22)	MD +0.11 (−0.02, 0.24)	0	Yes

Note: Michaelis 2024 was excluded as the highest-risk observational study (Newcastle–Ottawa Scale 5/9; see Figure S1 for domain justifications). These values were independently recalculated by the authors from the original extracted study-level data (DerSimonian–Laird random-effects) and are reported in full in the main text (Results, Sensitivity analyses subsection). Michaelis 2024 did not contribute to the ejaculatory-dysfunction pooled estimate reported in Table 2 of the main text; that estimate is therefore unaffected by this exclusion and is not repeated here.

Table S3. GRADE Summary of Findings

Outcome	Studies (n)	Relative Effect (95% CI)	Absolute Effect	Certainty	GRADE Downgrade Rationale
IPSS (post-operative)	5 (845)	MD −0.93 (−2.13 to 0.27)	No meaningful difference	■■■■ MODERATE	−1 Risk of bias: Only one partially randomized trial (open-label surgical design); four of five studies are observational. No downgrade for inconsistency (I^2 = 38%, acceptable heterogeneity). No downgrade for indirectness (direct head-to-head comparisons). No downgrade for imprecision (CI does not cross a clinically important threshold). No evidence of publication bias.
Qmax (mL/s)	4 (661)	MD −5.66 (−7.64 to −3.69)	LEP superior by ~5.7 mL/s	■■■■ MODERATE	−1 Risk of bias: Three of four studies are observational; single partially randomized trial with open-label design. No downgrade for inconsistency (I^2 = 16%, low heterogeneity). No downgrade for indirectness (direct comparisons, consistent populations). No downgrade for imprecision (narrow CI, large effect).
PVR (mL)	4 (661)	MD +11.96 (7.26 to 16.67)	LEP superior by ~12 mL	■■■■ MODERATE	−1 Risk of bias: Three of four studies observational. No downgrade for inconsistency (I^2 = 0%). No downgrade for indirectness. No downgrade for imprecision (CI excludes zero, clinically meaningful difference).
Residual prostate volume (mL)	2 (516)	MD +23.4 (13.3 to 33.5)	LEP superior by ~23 mL	■■■■ LOW	−1 Risk of bias: Both studies observational (Perez 2026 prospective comparative; WATER III partially randomized but open-label). −1 Imprecision: Only k = 2 studies; wide CI (13.3 to 33.5); high heterogeneity (I^2 = 80%).
Ejaculatory dysfunction	4 (461)	RR 0.19 (0.11 to 0.32)	Aquablation markedly lower risk	■■■■ MODERATE	−1 Risk of bias: Three of four studies observational; denominators subgroup-specific and partly approximated. No downgrade for inconsistency despite I^2 = 61% because effect direction is consistent across all studies and gland sizes. Large magnitude of effect (RR ~0.2) provides some reassurance. No downgrade for indirectness.
30-day hospital readmission	1 (2,104)	RR 4.84 (3.32 to 7.06)	Aquablation higher risk	■■■■ LOW	−1 Risk of bias: Administrative claims data with no clinical adjudication; coding accuracy dependent on billing practices. −1 Indirectness: No functional outcomes; propensity matching on administrative variables only; unable to control for prostate volume or surgeon experience.
90-day catheter re-insertion	1 (2,104)	RR 2.46 (1.51 to 4.00)	Aquablation higher risk	■■■■ LOW	−1 Risk of bias: Administrative claims data; no clinical validation of catheter re-insertion events. −1 Indirectness: Same limitations as readmission outcome; distinct data source from functional-outcome studies.
90-day urinary tract infection	1 (2,104)	RR 0.69 (0.52 to 0.92)	Aquablation lower risk	■■■■ LOW	−1 Risk of bias: Administrative claims data; UTI diagnosis based on coding, not microbiological confirmation. −1 Indirectness: Claims-based outcome distinct from clinical trial populations; no control for prophylactic antibiotic protocols.

Note: GRADE certainty ratings: ■■■■ HIGH, ■■■■ MODERATE, ■■■■ LOW, ■■■■ VERY LOW. Each downgrade is explicitly justified by GRADE domain. MD, mean difference (Aquablation − laser enucleation); RR, risk ratio (Aquablation vs laser enucleation); LEP, laser enucleation of the prostate; PVR, post-void residual.

Figure S1. Risk-of-Bias Assessment of Included Studies

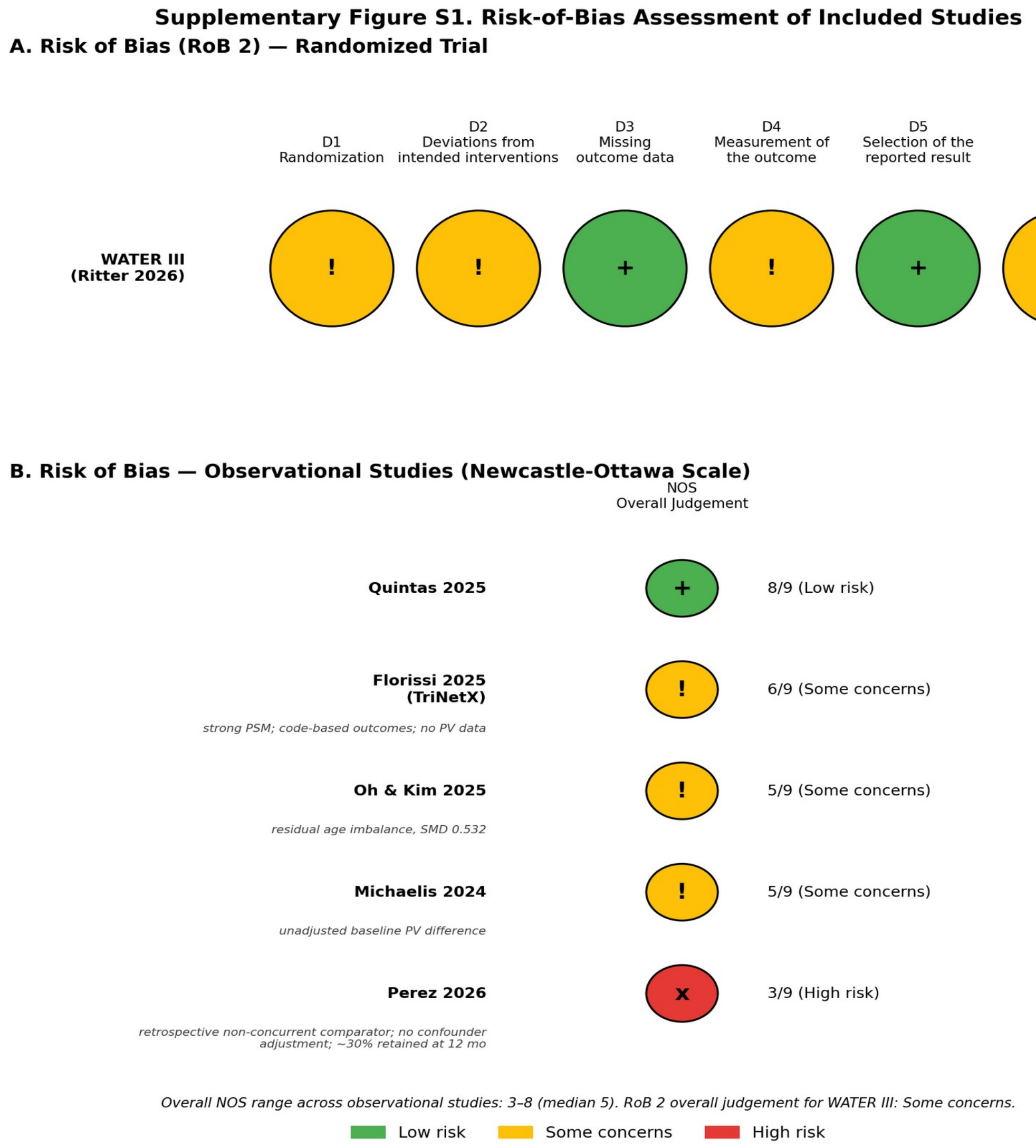

Figure S1. Risk-of-bias assessment of included studies. (A) Cochrane RoB 2 domain-level judgements for the partially randomized trial (WATER III), overall rated as some concerns, driven by the randomization process (combined randomized and non-randomized cohorts via Bayesian borrowing), deviations from intended interventions (open-label design), and measurement of the outcome (unblinded patient-reported IPSS); the missing-data and result-selection domains were rated low risk. (B) Newcastle-Ottawa Scale (NOS) scores for the five observational studies, with study-specific rationale for domain-level judgements: Quintas 2025 (8/9, low risk); Florissi 2025/TriNetX (6/9, some concerns — strong propensity-score matching but code-based outcomes, no prostate-volume data); Oh & Kim 2025 (5/9, some concerns — residual age imbalance, SMD 0.532); Michaelis 2024 (5/9, some concerns — unadjusted baseline prostate-volume difference); and Perez 2026 (3/9, high risk — retrospective non-concurrent comparator, no confounder adjustment, approximately 30% retained at 12 months). Overall NOS range across observational studies: 3–8 (median 5).
